# Biosynthesis of α-pyrones

**DOI:** 10.3762/bjoc.12.56

**Published:** 2016-03-24

**Authors:** Till F Schäberle

**Affiliations:** 1 Institute for Pharmaceutical Biology, University of Bonn, Nußallee 6, 53115 Bonn, Germany

**Keywords:** α-pyrones, biological activity, interconnecting ketosynthases, natural product, polyketides

## Abstract

The α-pyrone moiety is a structural feature found in a huge variety of biologically active metabolites. In recent times new insights into additional biosynthetic mechanisms, yielding in such six-membered unsaturated ester ring residues have been obtained. The purpose of this mini-review is to give a brief overview of α-pyrones and the mechanisms forming the basis of their natural synthesis. Especially the chain interconnecting enzymes, showing homology to ketosynthases which catalyze Claisen-like condensation reactions, will be presented.

## Introduction

**Figure 1:**
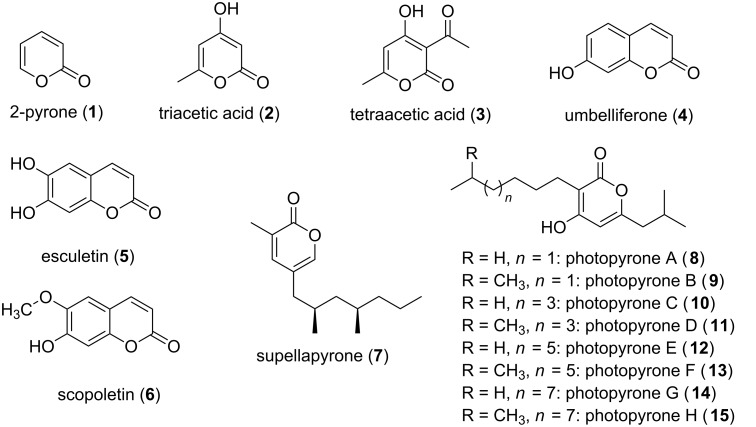
Selected monocyclic and monobenzo α-pyrone structures.

α-Pyrones (**1**, also 2-pyrones) represent a moiety widespread in nature (Figure 1). The motif of a six-membered cyclic unsaturated ester is present in a large number of natural products, and molecules containing α-pyrones can be found in all three kingdoms of life. Additionally α-pyrones, especially the structurally simple ones, i.e., triacetic acid lactone (**2**) and tetraacetic acid lactone (**3**) (Figure 1), represent widely exploited building blocks in synthetic chemistry. Examples are the syntheses of compounds like α-chymotrypsin, coumarins, pheromones, and solanopyrones [1]. Known biological functions reach from intermediates and end products in primary metabolism to signaling molecules and molecules which are applied for defense against competitors and predators. The biological activities these compounds exhibit is immense, including antimicrobial [2], antitumor [3–4], and cytotoxic activities [5]. Aflatoxins, produced by several *Aspergillus* species, are known to cause food poisoning due to their cytotoxic activity. They can regularly be found in improperly stored food, hence, entering the food supply chain [6]. Further coumarin derivatives, e.g., umbelliferone (**4**), esculetin (**5**), and scopoletin (**6**), are subject of investigation due to their pharmacological properties, i.e., anticancer effects (Figure 1) [7]. α-Pyrones have also been shown to be HIV protease [8–10] and selective COX-2 inhibitors [11–12], and further, signaling functions were attributed to them. Already in the 1990s an unusual dialkyl-substituted α-pyrone (supellapyrone, **7**) was detected to be the cockroach sex pheromone [13], and recently it was reported that so called photopyrones (**8**–**15**) act as signaling molecules in the cell–cell communication system of the bacterium *Photorhabdus luminescens* (Figure 1) [14].

Since the biological activities of α-pyrones are very diverse, these compounds are in the focus of synthetic chemists [15]. Hence, the phenomenal abundance of natural products and of chemically synthesized derivatives therefrom justifies several reviews, and comprehensive articles exist [1,16]. However, in the present review the diverse biosynthesis of α-pyrones will be the focus. Different mechanisms for the biosynthesis of these mostly polyketide-derived structures exist, thus it is assumed that the route towards α-pyrones has been developed several times in evolution. They can be built up by the catalytic activities of the different types of polyketide synthase (PKS) systems, and especially the final ring formation yielding in the α-pyrone moiety can be accomplished in different ways. The different biosynthetic routes towards an α-pyrone ring will be presented. The biosynthetic mechanisms to yield saturated lactones, like the statin drug lovastatin, which is in application for lowering cholesterol, will not be discussed.

## Review

### 1 Occurrence and activities

In this chapter special sub-types of α-pyrones will be described. The compounds are grouped into three categories depending on their structural features: (i) dibenzo-α-pyrones, (ii) monocyclic α-pyrones, and (iii) monobenzo-α-pyrones.

#### 1.1 Dibenzo-α-pyrones

**Figure 2:**
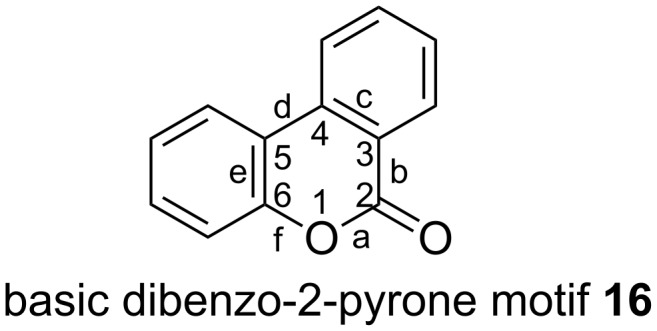
The basic core structure of dibenzo-α-pyrones.

Dibenzo-α-pyrones (**16**) harbor the α-pyrone moiety in the middle part and consist of three ring structures (Figure 2). Aromatic rings are fused to edge c and e of the central 2-pyrone, yielding the basic structure of **16**.

**Figure 3:**
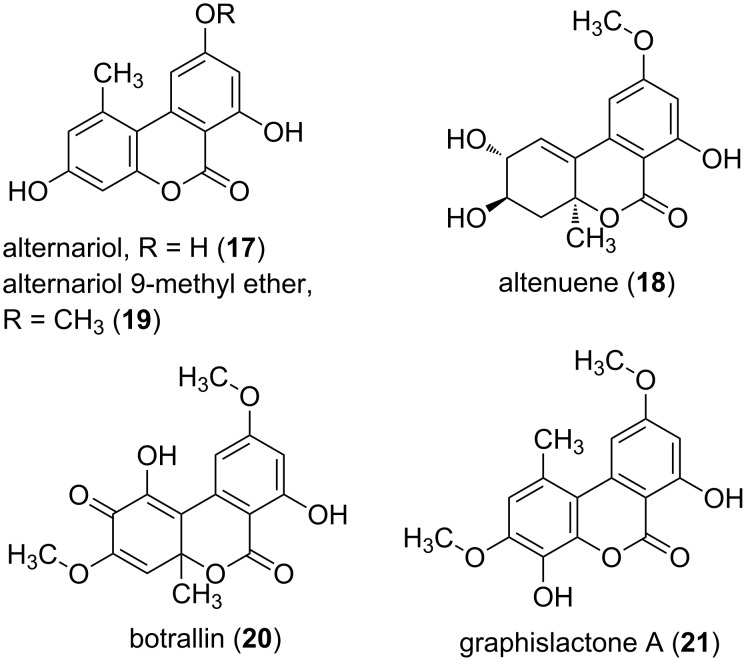
Selected dibenzo-α-pyrones.

Many dibenzo-α-pyrone-producing fungi have been described. However, it seems that they are mainly distributed in the *Alternaria* species and mycobionts. Especially endophytic fungi can be regarded as source organisms. Alternariol (**17**), altenuene (**18**), and alternariol 9-methyl ether (**19**) have been described from *Alternaria* sp. [17], botrallin (**20**) from *Hyalodendriella* sp. [18], and graphislactone A (**21**) from *Cephalosporium acremonium* IFB-E007 (Figure 3) [19]. These compounds show toxic effects in plants and animals. In addition, *Alternaria* spp. have been involved in the contamination of food, even in refrigerated stocks, since the fungi is able to grow also at low temperature. *Alternaria* spp. had also been linked to a poultry disease outbreak called poultry hemorrhagic syndrome. However, the main toxic effects seem to be linked to other toxins produced, e.g., the non pyrone metabolite tenuazonic acid [20]. Nevertheless, alternariol (**17**) and altenuene (**18**) were studied for their toxicity using different assays. Toxicity to *Artemia salina* larvae was examined by measuring the optical motility and resulted in IC_50_ values of 150 µg/mL [21]. A comparable result was obtained using the disk method of inoculation, whereby the IC_50_ values were 100 µg/mL for **17** and 375 µg/mL for **18** [22]. Further, alternariol (**17**) and derivatives were tested against L5178Y mouse lymphoma cells. Here **17** was the most active compound with an EC_50_ value of 1.7 μg/mL [23]. In another in vitro assay, this time a biochemical assay using protein kinase, the IC_50_ values were determined, and **17** inhibited 10 out of the 24 kinases tested. The results of the MTT and the kinase assay showed a similar pattern, and hence it was concluded that protein kinase inhibition should be one mechanism leading to the cytotoxicity of **17**. In a study using human colon carcinoma cells to elucidate the cell death mode and the pathways triggered by **17**, the induction of an apoptotic process was revealed. Further investigations showed that cell death was mediated through a mitochondria-dependent pathway [24]. In murine hepatoma cells it was shown that **17** and its methyl ether **19** interfere with the transcription factor and by inducing the so-called aryl hydrocarbon receptor, apoptosis is mediated by inducing cytochrome P450 1A1 [25]. For alternariol 9-methyl ether (**19**) and the graphislactone A (**21**) cytotoxic effects against the human cancer cell line SW1116 with IC_50_ values between 8.5 and 21 μg/mL were reported [26].

These toxic fungi-derived metabolites are often pathogenic to plants, and are therefore called phytotoxins. Phytotoxins are divided into host-specific and host non-specific toxins, whereby the here named *Alternaria*-derived dibenzo-α-pyrones **17**, **18**, and **19** represent host-specific phytotoxins [26].

**Figure 4:**
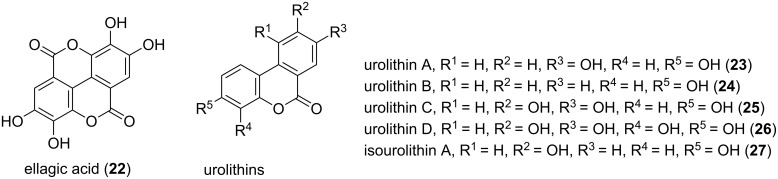
Structure of ellagic acid and of the urolithins, the latter metabolized from ellagic acid by intestinal bacteria.

Several dibenzo-α-pyrones have been isolated from plant parts. Purified from roots, bulbi, heartwood, or whole plant material, the origin of some plant-derived pyrones is not finally clarified, since the production by endophytic fungi cannot be excluded. Djalonensone was isolated from *Anthocleista djalonensis* (*Loganiaceae*) roots, but is identical to alternariol 9-methyl ether (the corresponding bioactivities are described above.) The latter was isolated from a series of fungi including endophytic species. Thus, the possibility that a fungus is the real producer cannot be ruled out. In addition, production by a fungus and modification of the metabolites by plant enzymes is also possible. Further α-pyrone plant secondary metabolites are ellagitannins and ellagic acid (**22**) [27] (Figure 4). These metabolites are important constituents of different foods, e.g., berries, nuts, medicinal plants and tisanes, as well as of grapes and oak-aged wines. These natural products are not absorbed in the intestinal tract; rather they are metabolized by intestinal bacteria, yielding so called urolithins (**23**–**27**, Figure 4). Therefore, it can be assumed that the urolithins are responsible for the biological activities related to the intake of ellagitannins by higher organisms. Such urolithins show different phenolic hydroxylation patterns and have been isolated from animal feces.

Concerning the activity urolithin A (**23**), urolithin B (**24**), and isourolithin A (**27**), all isolated from fruits of *Trapa natans* (water chestnut) showed antioxidant activity [28]. Testing urolithins A, B, C, D (**23–26**) in an assay using myelomonocytic HL-60 cells showed antioxidant activities for **23**, **25** and **26**. These three derivatives inhibited the reactive oxygen species (ROS)-dependent oxygenation of the non-fluorescent 2’,7’-dichlorodihydrofluorescein (DCFH) to the fluorescent 2’,7’-dichlorofluorescein (DCF) [29]. This antioxidant activity was also linked to anti-inflammatory effects by testing the in vivo effects of **23** in a carrageenan-induced paw edema assay. Oral administration of **23** to mice prior to carrageenan injection resulted in a significant decrease in paw edema, compared to the control group [30]. Further, weak antiallergic activity in the mM range was indicated for urolithin A (**23**), urolithin B (**24**), and isourolithin A (**27**), by testing the influences of these compounds on the activity of the enzyme hyaluronidase. The latter is involved in inflammation reactions. The authors isolated **23**, **24** and **27** from the feces of *Trogopterus xanthipes* (flying squirrel) by bioactivity-guided fractionation, and determined IC_50_ values for the pure compounds to be in the low mM range (1.33, 1.07 and 2.33 mM, respectively) [31]. Also estrogenic and antiestrogenic activities in a dose-dependent manner were shown for **23** and **24**. Thus, the authors suggested further research to evaluate the possible role of ellagitannins and ellagic acid as dietary “pro-phytoestrogens” [32].

**Figure 5:**
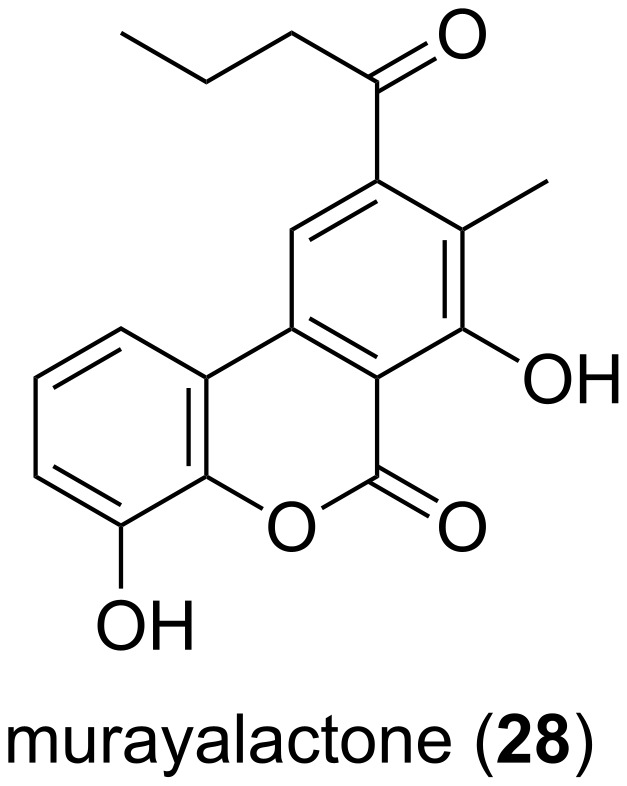
Structure of murayalactone, the only dibenzo-α-pyrone described from bacteria.

Even though many α-pyrones have been isolated from bacteria, only one dibenzo variant was described, i.e., murayalactone (**28**) isolated from *Streptomyces murayamaensis* (Figure 5) [33].

#### 1.2 Monocyclic α-pyrones

**Figure 6:**
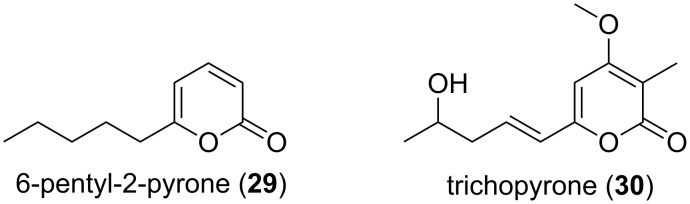
Structures of the 6-pentyl-2-pyrone (**29**) and of trichopyrone (**30**). Only **29** showed antifungal activity.

In addition to the aforementioned examples also the simplest α-pyrones show remarkable biological effects. Isolated from several fungi, e.g., *Trichoderma viride*, 6-pentyl-α-pyrone (**29**) showed antifungal activity against *Rhizoctonia cerealis*, *Gaeumannomyces graminis* and *Botrytis cinerea* (Figure 6) [34]. The structural related trichopyrone (**30**) instead showed no antimicrobial activity [35]. For compound **29** it was further revealed that it represents the prominent headspace volatile of *Trichoderma asperellum* IsmT5 [36]. Deeper investigation of the volatiles released by *Trichoderma* species revealed the complexity of the volatile mixture consisting of many derivatives [37]. Several alkylated and alkenylated α-pyrones with length variations in the side chain and different positions of olefinic double bonds were isolated in the headspace extracts and unambiguously assigned by comparison to authentic standards [37]. Co-cultivation experiments of *T. asperellum* and *Arabidopsis thaliana* without physical contact resulted in smaller but vital and robust plants. Therefore, **29** was applied to *A. thaliana*, and the growth and defense reactions were verified. *A. thaliana* pre-exposed to **29** showed significantly reduced symptoms when challenged with *B. cinerea* and *Alternaria brassicicola* [36].

**Figure 7:**
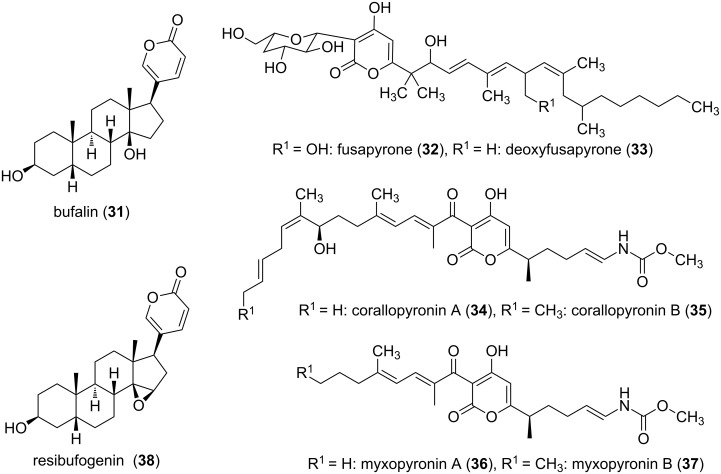
Selected monocyclic α-pyrones.

Beside the examples of simple substituted α-pyrone derivatives, such as triacetic acid lactone (**2**), tetraacetic acid lactone (**3**), and 6-pentyl-2-pyrone (**29**) also more complex systems, e.g., bufalin (**31**) [38], fusapyrones (**32,33**) [39], or the α-pyrone antibiotics corallopyronins (**34,35**) [40] and myxopyronins (**36,37**) [41], exist in the group of monocyclic α-pyrones (Figure 7).

The bufadienolides are an important group of steroids containing an α-pyrone moiety. The α-pyrone ring is here connected to a steroid nucleus, as exemplified in bufalin (**31**, Figure 7). These α-pyrones were detected in several plants, as well as in animals. The vast amount of derivatives shows also very diverse biological activities. The bufadienolides from succulent plants of the family Crassulaceae cause the symptoms of cardiac poisoning in animals. Animal sources are the name giving toad genus *Bufo* and others, e.g., *Photinus* (fireflies) and *Rhabdophis* (snake). The abundance of bufadienolides in some *Bufo* species is extremely high, and all together, over eighty derivatives have already been isolated, e.g., the epoxide-containing resibufogenin (**38**, Figure 7) was isolated from the Chinese toad skin extract drug Ch´an Su. It showed growth inhibition effects on human oral epidermoid carcinoma KB cells and murine leukemia MH-60 cells [42].

Testing the inhibitory effect of corallopyronin A (**34**) against various microorganisms revealed promising activity against Gram-positive bacteria, but no relevant effect on Gram-negative bacteria (only at concentrations >100 µg/mL activity was observed). Against *Staphylococcus aureus* a MIC of 0.097 µg/mL and against *Bacillus megaterium* of 0.39 µg/mL was obtained [40]. Myxopyronin B (**37**), the most active derivative of the myxopyronins, showed comparable activities, e.g., MIC of 0.3 and 0.8 µg/mL against *S. aureus* and *B. megaterium*, respectively [43]. In addition corallopyronin A was also tested successfully using an in vivo mouse model for the treatment of infections with filarial nematodes [44]. Such antibiotics produced by heterotroph bacteria, e.g., marine and terrestrial myxobacteria which can feed on other bacteria, are suggested as predatory weapons to paralyze and kill their prey [45–46].

**Figure 8:**
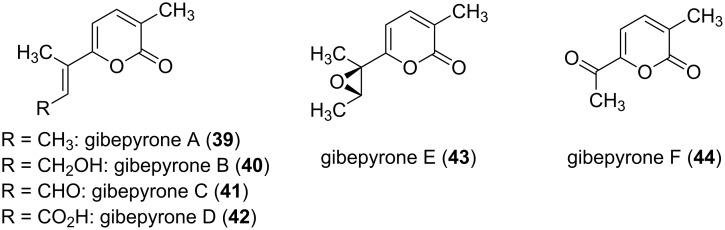
Structures of the gibepyrones A–F.

Fusapyrone (**32**) and the derivative deoxyfusapyrone (**33**) had been isolated from *Fusarium semitectum* [39]. These compounds show considerable antifungal activity, e.g., a minimum inhibitory concentration against *Botrytis cinerea*, *Aspergillus parasiticus*, and *Penicillium brevi-compactum* in the range of 0.78–6.25 µg/mL [47]. Testing the zootoxicity of **32** and **33**, using brine shrimp assays, revealed that only approximately 50-fold higher concentrations had a negative effect. Therefore, it was concluded that these compounds might be used together with biocontrol yeasts to control crop diseases which can occur while storing the crops [47]. From another strain of this fungal genus, i.e., *Fusarium fujikuroi*, the gibepyrones A–F (**39**–**44**) were isolated (Figure 8) [48]. The activity of these compounds was tested against bacterial and fungal strains. However, the activities were extremely low, e.g. gibepyrone A inhibited *B. subtilis* and *S. cerevisiae* at 100 µg/mL.

**Figure 9:**
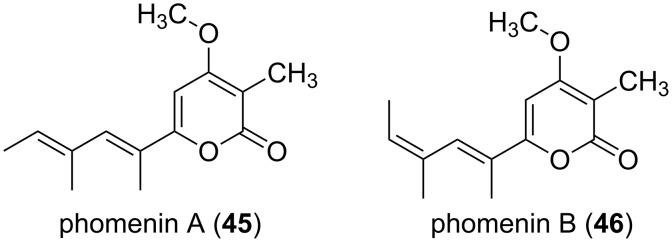
Structures of the phomenins A and B.

The diastereomeric pair of phomenin A (**45**) and phomenin B (**46**) was isolated from the phytopathogenic fungus *Phoma tracheiphila*,[49] and from *Alternaria infectoria* (Figure 9) [50]. Further, the same compound **45** was isolated from *Leptosphaeria maculans* and named phomapyrone A, as well as from the mediterranean ascoglossan mollusc *Ercolania funereal*, described as cyercene [51]. Phomenin A displayed phytotoxicity at a concentration of 100 µg/mL. Chemical synthesis approaches enabled then to investigate many more α-pyrone derivatives for their antimicrobial and cytotoxic properties [2].

**Figure 10:**
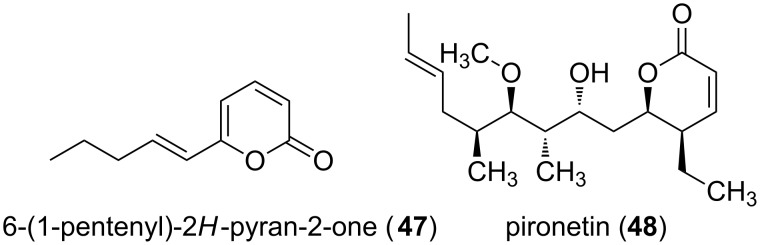
Structures of monocyclic α-pyrones showing pheromone (**47**) and antitumor activity (**48**), respectively.

The volatile α-pyrone 5-(2,4-dimethylheptyl)-3-methyl-2*H*-pyran-2-one (**7**, Figure 1), also named supellapyrone) is used by female brownbanded cockroaches to attract males [13]. It is known that cockroaches use pheromones in many aspects of influencing interacting behavior between individuals. Hence, such volatiles are used in courtship behavior to find mating partners. Also another α-pyrone fulfilling pheromone function in insects is known, i.e., the queen recognition pheromone of the red imported fire ant, 6-(1-pentenyl)-2*H*-pyran-2-one (**47**, Figure 10) [52].

Also antitumor activities of α-pyrones had been shown. Thus, pironetin (**47**, Figure 10) induced apoptosis in a dose- and time-dependent manner, and tubulin assembly was inhibited in vitro [53]. The natural product was isolated from *Streptomyces* sp. NK10958 [54], and its biosynthesis was investigated using various ^13^C-labeled precursors [55]. Hence, it was concluded that beside four acetate units also two propionate units and one butyrate unit form the backbone, while the *O*-methylation is S-adenosyl-methionine dependent.

**Figure 11:**
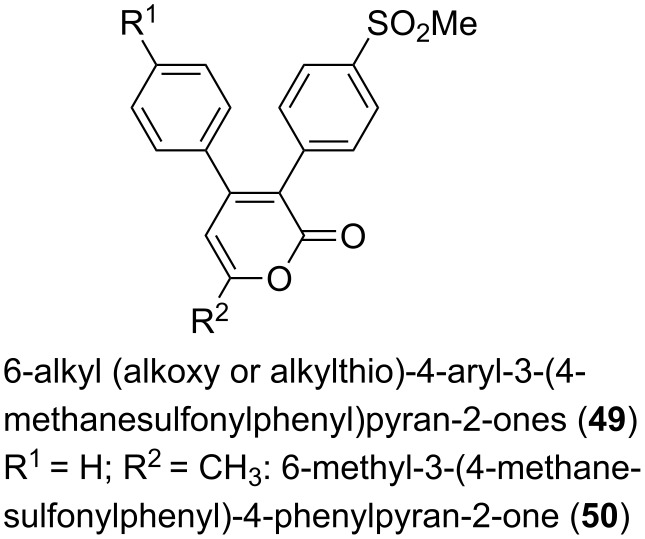
Structures of 6-alkyl (alkoxy or alkylthio)-4-aryl-3-(4-methanesulfonylphenyl)pyrones.

Also cyclooxygenase-2 (COX-2) inhibitors are an interesting target of research, due to the fact that the progression of Alzheimer’s disease was slowed down by using anti-inflammatory drugs. Thus, selective COX-2 inhibitors, anti-inflammatory compounds themselves, might have beneficial effects in vivo. Several derivatives of 6-alkyl (alkoxy or alkylthio)-4-aryl-3-(4-methanesulfonylphenyl)pyrones **49** had been synthesized to get insights into structure activity relationships, whereby 6-methyl-3-(4-methanesulfonylphenyl)-4-phenylpyran-2-one (**50**) showed the best combination of inhibitory concentration and selectivity (IC_50_ = 0.68 µM, SI = 904; Figure 11) [56].

**Figure 12:**
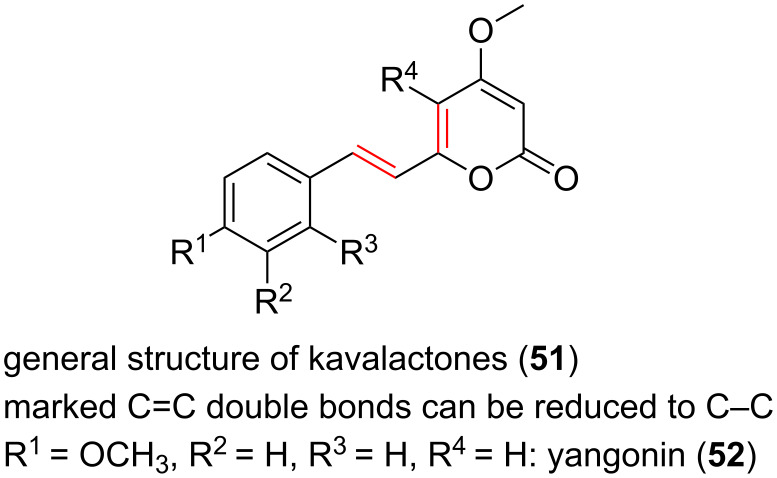
Structures of kavalactones.

A further group of compounds are the kavalactones **51** (Figure 12), e.g., yangonin (**52**, Figure 12), which have been isolated from *Piper methysticum* [57]. At various regions of the Pacific Ocean the roots of the plant have been used for a long time to produce a drink with sedative and anesthetic properties. The α-pyrones responsible for the influence on the nervous system have a wide variety of effects including amnestic, analgesic, anticonvulsant, anxiolytic, nootropic, and sedative/hypnotic activities [58].

**Figure 13:**
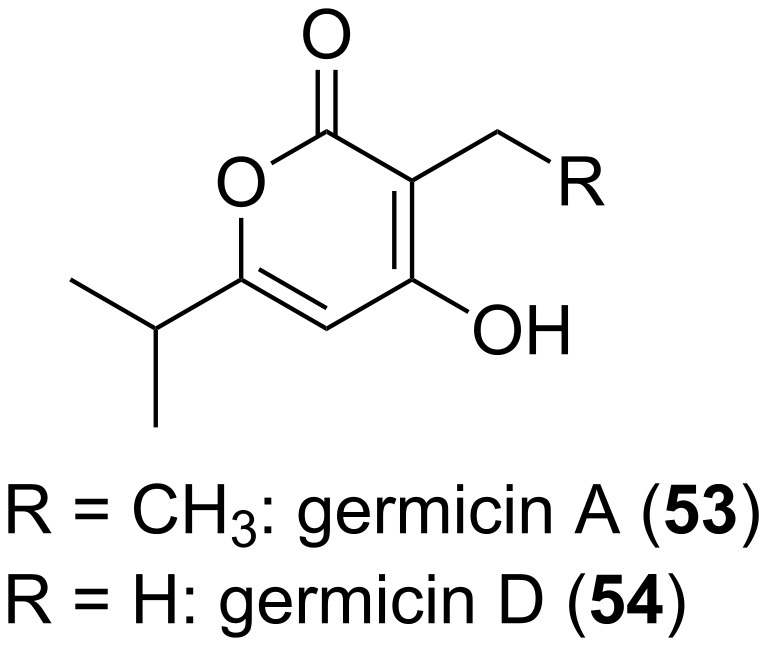
Strutures of germicins.

Highly active α-pyrones, i.e., germicidins (**53**, **54**, Figure 13), were isolated from *Streptomyces viridochromogenes* NRRL B-1551, whereby the compounds had been detected in the supernatant of germinated spores, as well as in the supernatant of the submerged culture [59]. The excretion of these compounds prevents the germination of the spores too close to the parent culture. Germination of *S. viridochromogenes* NRRL B-1551 spores is inhibited at pM concentrations, i.e., 200 pM (40 pg/mL). A comparable effect was also observed by applying **53** and **54** to seeds, however, only at much higher concentrations. Germination of *Lepidium sativum* (garden cress) seeds was clearly retarded. An additional in vitro effect was inhibition of porcine Na^+^/K^+^-activated ATPase. Germicidin was the first known autoregulative inhibitor of spore germination in the genus *Streptomyces* [59]. Influence on plant germination was also shown for further lactones. An inhibiting effect was proven for 3,4-dimethylpentan-4-olid from the plant pathogenic fungus *Hymenoscyphus pseudoalbidus*, which inhibited germination of *Fraxinus excelsior* (European ash) seeds [60]. In contrast, 3-methyl-2*H*-furo[2,3-*c*]pyran-2-one, a component of smoke derived from burning plant material, promotes seed germination [61].

**Figure 14:**
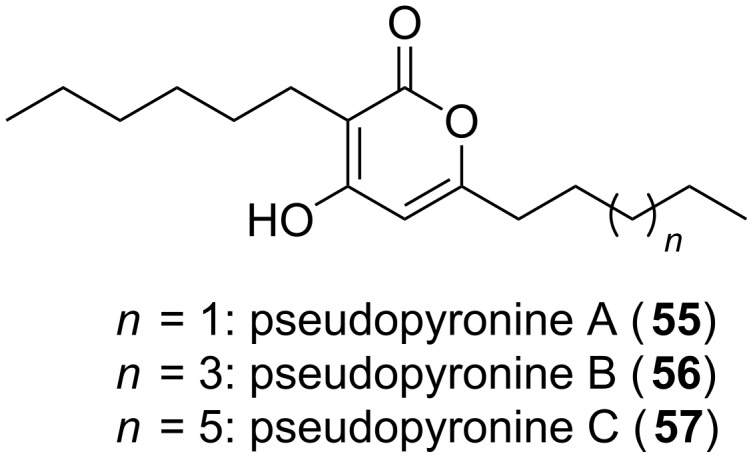
Structures of the pseudopyronines.

Recently, a further regulatory function for α-pyrones within bacteria was discovered. The so called photopyrones (**8**–**15**, Figure 1) represent extracellular signals involved in cell–cell communication [14]. *Photorhabdus luminescens*, an entomopathogenic bacterium species, excretes these molecules, and binding of the latter to the respective receptor, i.e., the PluR protein, leads to the activation of the *Photorhabdus* clumping factor (PCF) operon (*pcfABCDEF*). The phenotypic change observed due to PCF expression was cell clumping, which in turn contributed to insect toxicity [14]. Structurally related are the pseudopyronines A (**55**), B (**56**), and C (**57**, Figure 14), which have been isolated from different *Pseudomonas* strains [62–63]. Compounds **55** and **56** had been initially tested positive for antimycobacterial and antiparasitic activities and both inhibited fatty acid biosynthesis [62]. The new derivative **57**, possessing a longer eastern acyl moiety, was identified in *Pseudomonas* sp. GM30, and it was subsequently proven by heterologous expression experiments with ketosynthase which is responsible for the biosynthesis of these derivatives [63].

#### 1.3 Monobenzo-α-pyrones

**Figure 15:**
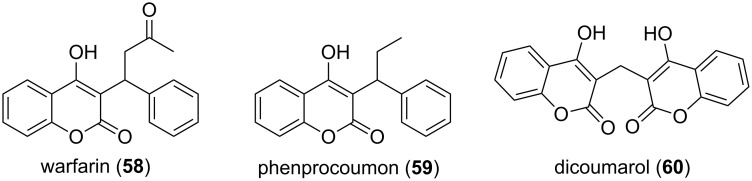
The structures of the monobenzo-α-pyrone anticoagulant drugs warfarin and phenprocoumon.

Synthetic derivatives of the natural product 4-hydroxycoumarin are widely used as anticoagulant drugs. Warfarin (**58**, Figure 15) – initially introduced as a pesticide against rats and mice – is the most described oral anticoagulant drug in North America. The derivative phenprocoumon (**59**, Figure 15) is the most commonly used anticoagulant in Germany. Phenprocoumon was further identified as a lead template with HIV protease inhibitory activity, i.e., *K*_i_ = 1 µM [64]. However, the prototype of these anticoagulant drugs was dicoumarol (**60**), which was in use until it was replaced by other derivatives, e.g., **58** and **59** [65].

**Figure 16:**
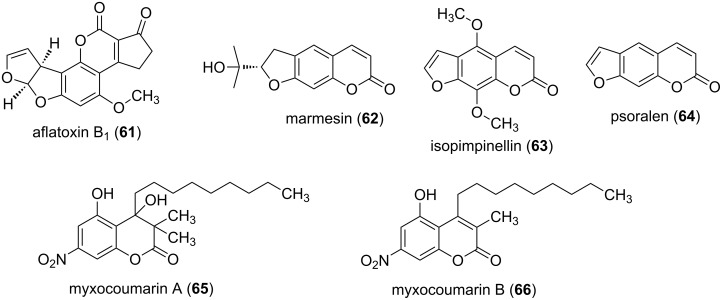
Structures of selected monobenzo-α-pyrones.

Aflatoxins are poisonous and cancer-causing monobenzo-α-pyrones [6]. Several derivatives exist, whereby aflatoxin B_1_ (**61**, Figure 16) represents the most poisonous compound. Usually these toxins are ingested, but **61** can also permeate through the skin. The aflatoxins are PKS-derived molecules which undergo an extreme rearrangement [66]. The cytotoxic effects of the coumarin derivatives umbelliferone (**4**, Figure 1), esculetin (**5**, Figure 1), and scopoletin (**6**, Figure 1) are subject of anticancer research [67]. Marmesin (**62**) was first isolated from the fruits of *Ammi majus* [67], and is currently under investigation as an agent for the treatment of angiogenesis-related diseases, e.g., cancer [68]. A structurally related compound, i.e., isopimpinellin (**63**), was also first isolated from fruits of *Ammi majus* [69]. It was shown that **63** blocks DNA adduct formation and skin tumor initiation in mice [70]. Psoralen (**64**), isolated from plants, e.g., *Ficus carica*, had been used against skin diseases due to its mutagenic effect [71].

Bacterial monobenzo-α-pyrones were isolated from the myxobacterium *Stigmatella aurantiaca* MYX-030. Myxocoumarins A (**65**) and B (**66**) were identified, and **65** was tested for antifungal activity [72]. It showed a promising activity against agronomically important pathogens, e.g., complete inhibition of *Magnaporthe grisea* and *Phaeosphaeria nodorum* at 67 µg/mL, and *Botrytis cinerea* was inhibited at 200 µg/mL.

### 2 Biosynthesis

Even though the α-pyrones possessing interesting activities were in the focus of chemical synthesis approaches for a long time, for most of them the clarification of the biosynthesis remained unknown for many years.

**Figure 17:**
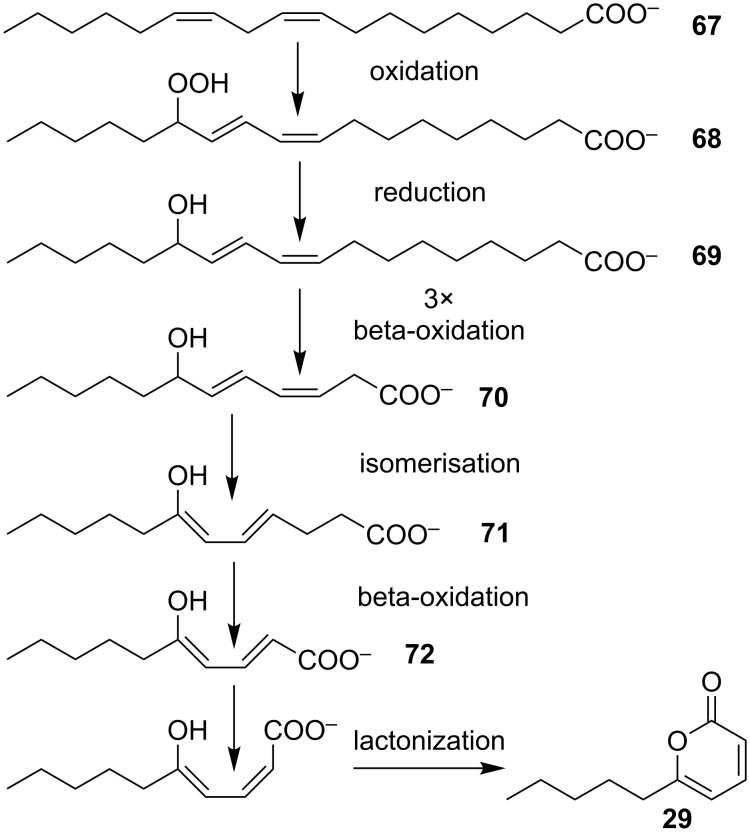
Hypothetical pathway of **29** generation from linoleic acid [34].

An early example for a biosynthetic hypothesis is the biosynthesis of the simple 6-pentyl-α-pyrone (**29**), which was hypothesized to start with the C-18 linoleic acid. This acid is then shortened by β-oxidation reactions to a C-10 intermediate, i.e., 5-hydroxy-2,4-decenoic acid (**72**), which undergoes lactonization to yield **29** (Figure 17) [34]. This hypothesis is based on the fact that feeding studies with *Trichoderma harzianum* and *T. viride* using [U-^14^C]linoleic acid or [5-^14^C]sodium mevalonate revealed the incorporation of these labelled compounds into 6-pentyl-α-pyrone (**29**). Labelled sodium mevalonate was used to test for the possible link between the isoprenic pathway and biosynthesis of **29**. The experiments revealed that the incorporation of labelled linoleic acid reached within the first 24 hours 18-fold higher ratios than labelled sodium mevalonate. Therefore, the authors suggested that β-oxidation of linoleic acid is a probable main step in the biosynthetic pathway of **29** in *Trichoderma* species [34]. The incorporation of labelled sodium mevalonate is hypothesized to be due to degradation to acetate with following polymerization to fatty acids [34].

**Figure 18:**
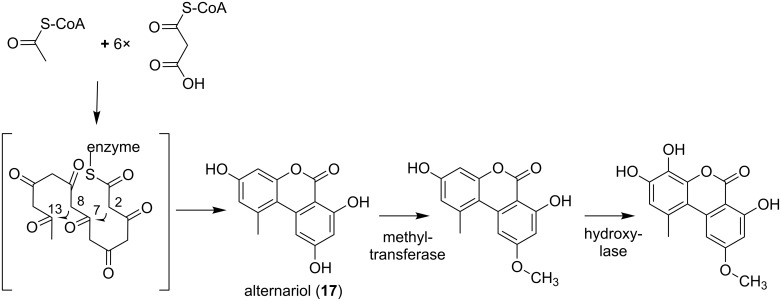
Proposed biosynthetic pathway of alternariol (modified from [77]). Malonyl-CoA building blocks are applied to build up the enzyme-bound polyketide chain. Cyclization between C-2, C-7 and C-8, C-13, as well as lactonization takes place, resulting in alternariol (**17**). Subsequently, a methylation and a hydroxylation reaction occur, catalyzed by the respective enzymes.

Now, it is generally accepted that most α-pyrones are synthesized via the polyketide pathway. Solely for plant-derived ellagitannins another biosynthetic origin was described. Via the shikimate pathway gallic acid is generated, which represents the precursor in ellagitannin biosynthesis [73]. The ellagitannins can then be hydrolyzed to ellagic acid (**22**), and subsequently converted to urolithins (**23**–**27**). In microorganisms the PKS-derived origin was independently postulated for numerous compounds. The polyketide biosynthesis has much in common with fatty acid biosynthesis: The mechanisms of chain elongation resemble each other, and simple building blocks, e.g., acetyl-CoA and malonyl-CoA, are used to build up the molecule [74]. In general both, polyketides and fatty acids are assembled by repeating Claisen-condensations between an activated acyl-starter unit and malonyl-CoA-derived extender units. This process is catalyzed by the concerted action of a ketosynthase (KS), an acyltransferase (AT), and either a phosphopantetheinylated acyl carrier protein (ACP), or CoA to which the nascent chain is attached. After each elongation step the β-keto functionality can be reduced by further enzymes involved. In fatty acid biosynthesis usually a complete reductive cycle takes place, i.e., a ketoreductase (KR) generates a hydroxy group, a dehydratase (DH) reduces to an alkene double bond, and an enoyl reductase (ER) yields a completely saturated acyl-backbone. These reductive steps are optional in PKS biosynthesis, and considering the pyrone ring formation, an unsaturated PKS chain residue attached to the carrier is essential. This general PKS catalyzed mechanism is accomplished by different enzymatic machineries. In the following section the three PKS types which can be responsible for the biosynthesis of the polyketide chain are described. A strong indication was that in the genome of the alternariol producer *Alternaria alternate* two PKS genes, i.e., *pksJ* and *pksH*, had been identified, whose expression pattern was in correlation with alternariol (**17**) production [75]. Mutant strains with downregulated expression level for these PKSI systems were constructed and suggested that PksJ is the PKS required for the biosynthesis of **17**. PksH downregulation affected *pksJ* expression and in that way influenced biosynthesis of **17** as well. The initially postulated biosynthesis via norlichexanthone was ruled out by incorporation studies in *Alternaria tenuis* using [1-^13^C, ^18^O_2_]-labeled acetate. This resulted in high incorporation of acetate-derived oxygen into all the oxygen-bearing carbons [76]. A proposed biosynthetic pathway of **17** [77] (by aromatization of a polyketide), and of derivatives (by post-PKS reactions) is shown in Figure 18. The authors suggested that seven malonyl-CoA building blocks are connected via Claisen-condensation reactions, followed by aldol-type cyclizations between C-2 and C-7, as well as between C-8 and C-13. The subsequent lactonization yields alternariol (**17**). However, it can be assumed that the starter molecule should be acetyl-CoA. Through subsequent chain elongation by six malonyl-CoA extender units the linear chain is assembled. It has to be mentioned, that there is still an ongoing debate about the real alternariol-producing PKS in *A. alternate*, but the building blocks and the general mechanism are accepted [78].

#### 2.1 Biosynthesis by PKSI systems

**Figure 19:**
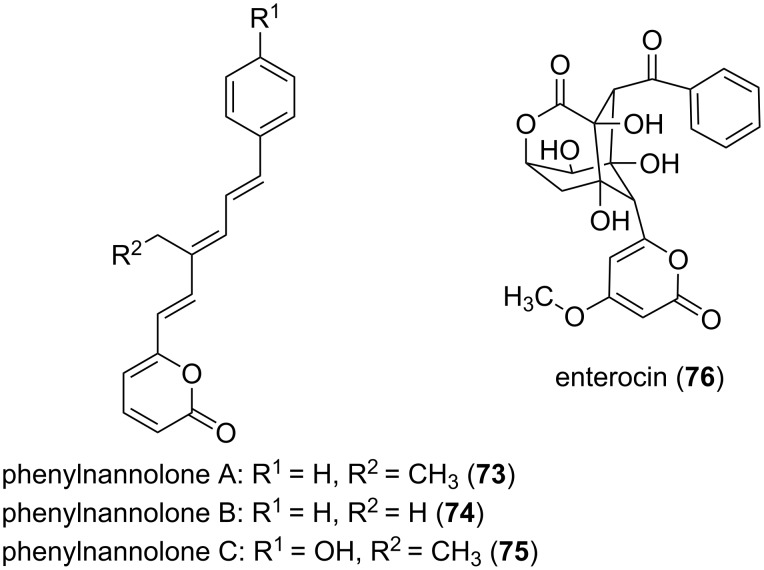
Structures of phenylnannolones and of enterocin, both biosynthesized via polyketide synthase systems.

**Figure 20:**
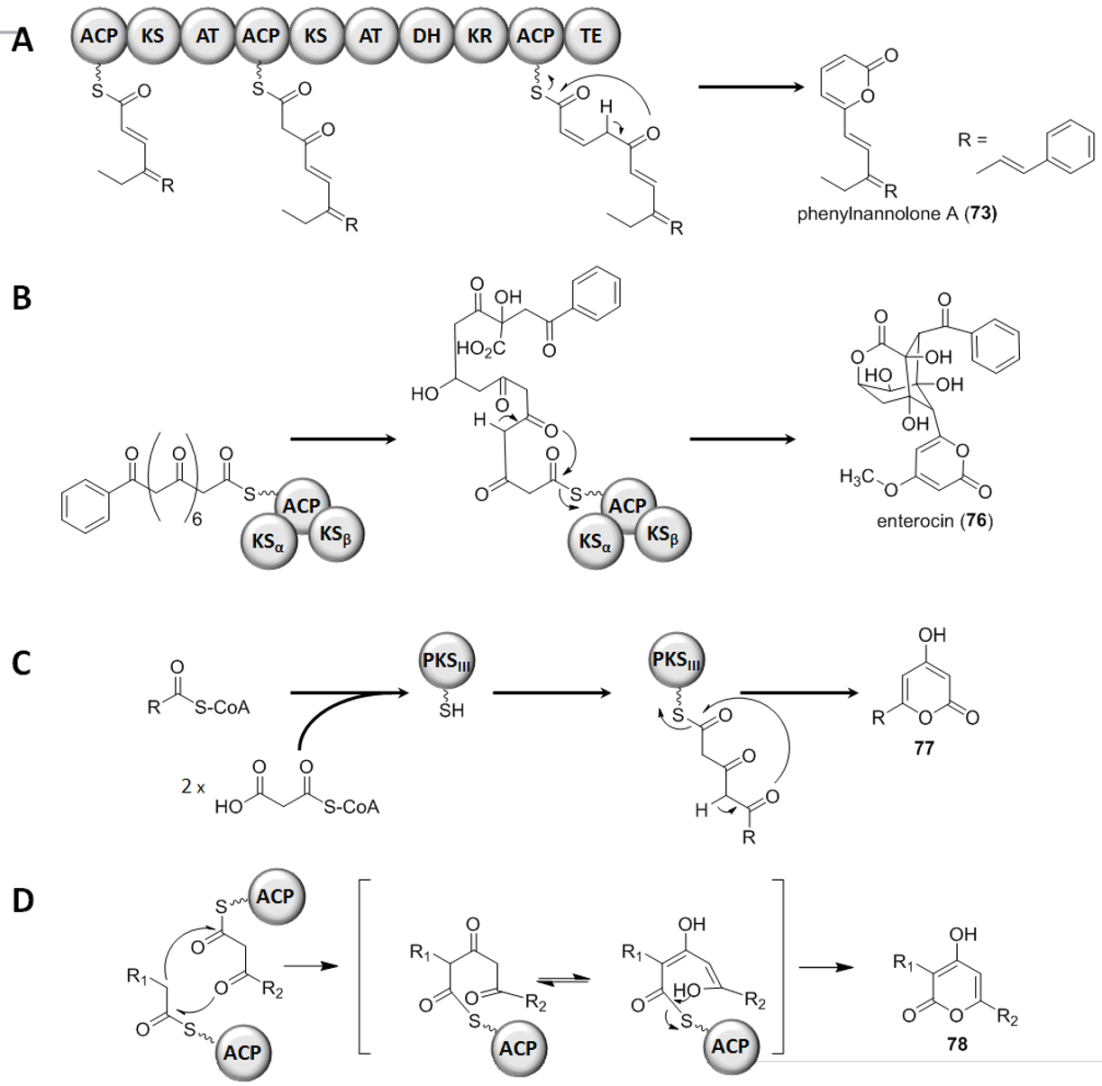
Pyrone ring formation. Examples for the three types of PKS systems are shown in A–C. In D the mechanism catalyzed by a free-standing ketosynthase is depicted. Herein the keto–enol tautomerism is shown. A) Polyketide synthase (PKS) type I: The end part of the phenylnannolone A biosynthesis is given. The ACP-tethered nascent chain gets elongated by the incorporation of acetate units. The corresponding reductive domains (ketoreductase, KR; and dehydratase, DH) reduce the β-keto group to a *cis* double bond. The chain is then released from the assembly line through pyrone ring formation catalyzed by the thioesterase (TE) domain, resulting in **73**. B) PKS type II: The precursor of the enterocin biosynthesis, comprising the uncommon benzoate starter unit, is shown attached to the ACP domain, which forms a complex with the KS_α_ and the KS_β_ domain. Modification, rearrangement and lactonization of this bound precursor yield enterocin (**77**). C) PKS type III: The starter molecule, e.g., a CoA-activated fatty acid, gets loaded to the PKS III enzyme. Two rounds of chain elongation via malonyl-CoA take place before the molecule is released by pyrone ring formation, resulting in **77**. D) The two ACP-tethered chains are interconnected by the catalytic activity of a free-standing KS. In the second step the lactonization takes place, facilitated by the keto–enol tautomerism. Thereby the α-pyrone **78** is formed.

The biosynthesis of an α-pyrone by a modular PKSI system will be showcased using the phenylnannolone (**73**–**75**, Figure 19) pathway (Figure 20) [79]. The aromatic starter is cinnamic acid, which is elongated by a butyrate moiety. Subsequently three further elongation steps, this time using malonate as extender units, follow. This results in the incorporation of acetate units via Claisen-condensation reactions. The reductive domains, i.e., ketoreductase (KR) and dehydration (DH) domains, present in the distinct modules reduce the keto group in a stepwise manner to the hydroxy group and the C=C double bond. Subsequently, the KR present in the terminal module catalyzes the reduction of the β-keto group to an L-hydroxy group. This hydroxy is then further reduced by the catalytic activity of the DH in the terminal module, which results in a *cis*-configured double bond. Through the formation of the *cis* double bond the sterical arrangement of the nascent chain favors the lactone ring closure which results in the α-pyrone moiety. Hence, the polyketide is released from the assembly line, whereby the thioesterase (TE) domain catalyzes the ring-closure and therewith also the off-loading from the PKSI system [79]. A comparable mechanism, in which a TE is involved in off-loading the nascent chain from the PKS assembly line by lactonization, was described for other natural products, e.g., the isochromanone ring formation for the ajudazols A and B in *Chondromyces crocatus* Cm c5 [80].

#### 2.2 Biosynthesis by PKSII systems

In the type II PKS-catalyzed biosynthesis, the subunit type of such megaenzyme systems, the starter molecule and the extender units, mostly malonate molecules, are assembled at the same ACP. A lactonization at the ACP-bound terminus yields the pyrone ring. As an example the enterocin (**76**, Figure 19) biosynthesis will be regarded (Figure 20). In the marine bacterium *Streptomyces maritimus* a gene cluster corresponding to enterocin (*enc*) biosynthesis was identified [81]. The minimal *enc* PKS, EncABC, is encoded by a set of genes architecturally similar to most other type II PKS clusters. EncA represents the KSα, EncB the KSβ, and EncC the ACP domain. First, an uncommon benzoate starter unit gets elongated by seven malonate molecules. This nascent carbon chain undergoes a rare Favorskii-like rearrangement and lactonization to yield the polyketide **76**.

#### 2.3 Biosynthesis by PKSIII systems

Type III PKSs are relatively small molecules, since in contrast to the PKSs of type I and II they solely consist of a single ketosynthase. A single KS connects the CoA-bound starter and extender units; and also in this system the final lactonization of the peptide-bound polyketide chain results in the pyrone ring. Type III systems synthesize a variety of aromatic polyketides. First discovered in plants, later PKS III systems have also been described in fungi and bacteria. BpsA (for *Bacillus* pyrone synthase) was analyzed in vivo and in vitro [82]. These experiments revealed BpsA to be indeed the enzyme responsible for the synthesis of triketide pyrones. The substrates used by BpsA are long-chain fatty acyl-CoAs and malonyl-CoAs – either as starter or as elongation building blocks, respectively (Figure 20). Generating *B. subtilis* mutant strains, overexpressing the *bpsA* gene, yielded in triketide pyrenes. Once the adjacent gene *bpsB*, the latter coding for a methyltransferase, was co-overexpressed, the methylated variants, i.e., triketide pyrone methyl ethers, were synthesized. The pyrone-forming activity of BpsA was also proven in vitro, using heterologously expressed protein. Thereby, the chain length of the acyl residue had only minor influence on the pyrone formation, since many substrates had been accepted. This could be expected, since the α-pyrone formation takes place at the enzyme-tethered end of the nascent chain, resulting in off-loading.

#### 2.4 Biosynthesis by free-standing ketosynthases

**Figure 21:**
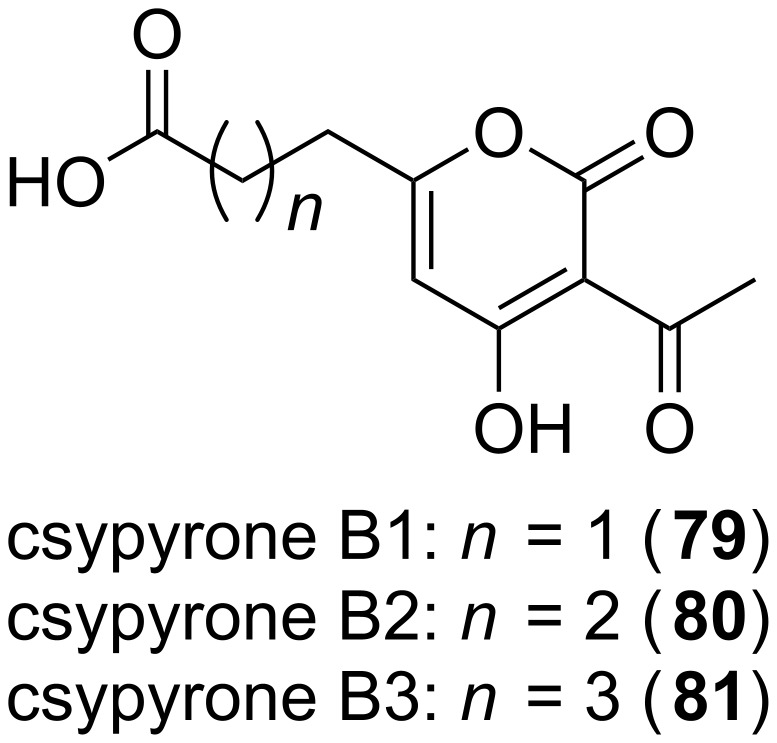
Structures of csypyrones.

In contrast to the α-pyrone formation by intramolecular cyclization reactions, also the condensation of two polyketide chains can result in a pyrone ring. Such a mechanism was indicated by feeding experiments for the antibiotically active compounds **36** [83] and **34** [84]. The resulting labeling pattern clearly showed that the central α-pyrone ring of the molecule was not the result of a usual intramolecular reaction. Rather, an interconnection of two independent chains should form the central ring structure. In addition further molecules, e.g., photopyrones (**8**–**15**) from *Photorhabdus luminescens* are synthesized by such a head-to-head condensation of two acyl moieties [60]. Also the csypyrones (**79**–**81**, Figure 21), first reported from *Aspergillus oryzae*, are composed of two independent chains which are interconnected thereafter [85]. Recently, the biosynthetic origin of the pseudopyronines A (**55**) and B (**56**) in *Pseudomonas putida* BW11M1 was clarified – and again two chains are fused to yield the final products [86]. Thus, it can be assumed that this mechanism is exemplified quite often in natural products. Therefore, in the next paragraph the chain interconnecting mechanism will be described.

For α-pyrone antibiotics, the corallopyronin and myxopyronin derivatives, free-standing KSs encoded in the respective cluster, i.e., CorB and MxnB, were suggested as the chain-interconnecting enzymes [84,87]. These enzymes have now been investigated in detail.

In vitro assays using NAC thioesters of the western and eastern chains in the biosynthesis of **36** [88], as well as simplified substrate mimics of both antibiotics [88–89] provided experimental evidence that the free-standing ketosynthases are responsible for the α-pyrone ring formation. In both publications non-enzymatic condensation was ruled out, since in the absence of the respective protein no product formation was detectable. For MxnB it was further shown that in vitro conditions can be optimized by applying carrier-protein-bound substrates instead of the SNAC-coupled substrates, i.e., this resulted in a 12-fold increase of product formation. This is an additional hint that protein–protein interactions represent an important factor in PKS systems. Further, it seemed that the carrier proteins conferred specificity for α-pyrone ring formation, since once the carrier proteins were primed in each case with the other substrate (mimic), the production rate decreased significantly. However, a certain degree of flexibility in α-pyrone ring formation was proven by the in vitro experiments using the ketosynthases CorB and MxnB. In addition, the substrate specificity was analyzed in vivo in a mutasynthesis study employing a *Myxococcus fulvus* mutant unable to biosynthesize the western chain. This study revealed that MxnB is capable of condensing a wide variety of activated synthetic western chains with the carrier protein bound native eastern chain [90].

**Figure 22:**
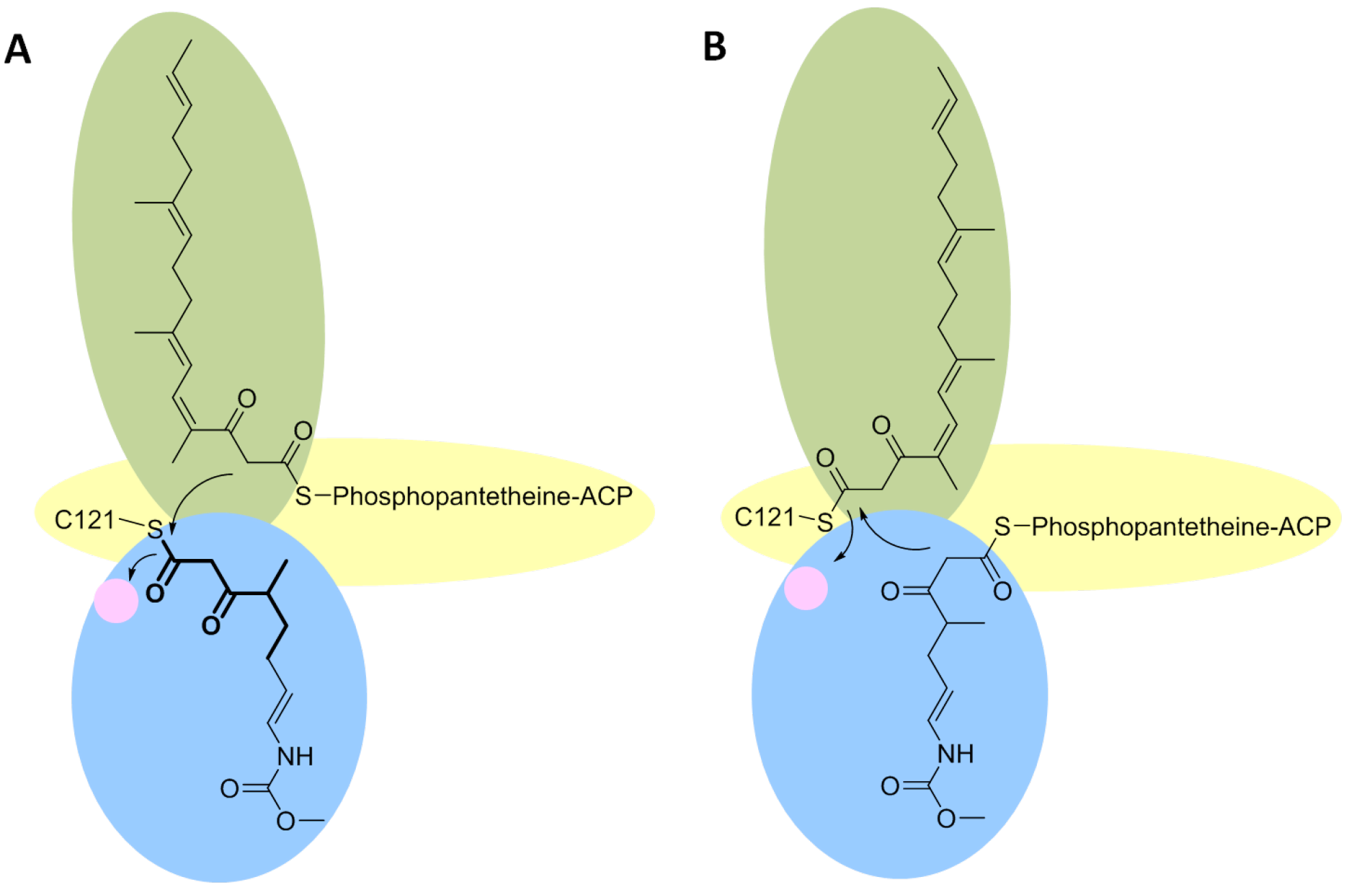
Schematic drawing of the T-shaped catalytic cavities of the related enzymes CorB and MxnB. The two cavities, each harboring one chain are depicted in green and blue, respectively. The phosphopantetheine arm of the ACP reaches into the T-shaped catalytic cavity through a third hydrophobic channel. The oxyanion hole is highlighted by a pink circle. In that way the two chains are positioned face to face. A) Transacylation of the eastern chain to C_121_ of CorB. The simplified mimic of the eastern chain (shown in bold) was placed into the active site on the basis of its unbiased (F_0_–F_c_)-difference electron density. The remaining portion of the eastern chain was modeled into the cavity. B) Transacylation of the western chain to the catalytic C_121_ of MxnB. In vitro experiments assaying MxnB together with substrate mimics indicate the transacylation of the western chain as the natural mechanism. It can be assumed that different chains alter the binding preferences for CorB and MxnB.

**Figure 23:**
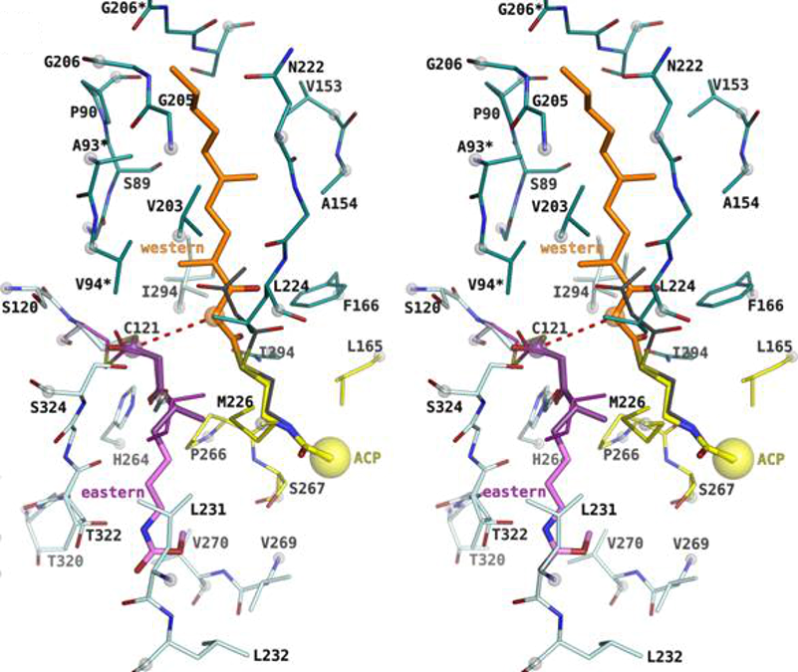
Stereo representation of the CorB binding situation (modified from [89]). The substrate mimic (dark violet) was placed into the active site on the basis of its unbiased (F_0_–F_c_)-difference electron density and the remaining portion of the eastern chain (light magenta) was modeled into the cavity. The western chain was modeled into the proximal cavity on the basis of a homologue α-pyrone synthase using the pantotheine entity as an anchor point.

The two proposed mechanism for CorB and MxnB closely resembles each other, but certain differences have also been proposed, as will be discussed here. First, one chain is transferred and covalently linked to the active-site cysteine. This results in an activation of the cysteine-tethered chain. In the second step, the other chain is placed into the proximal cavity, orienting the α-carbon in a position suitable for the nucleophilic attack by the cysteine-tethered, activated chain. Thereby, the second chain is still attached to the ACP, the phosphopantetheine residue reaching into the T-shaped catalytic cavity, enabling the placement of the two chains in opposite directions (Figure 22 and Figure 23). In that way a nucleophilic attack of the enzyme-bound chain onto the carbonyl carbon of the ACP-tethered chain is facilitated. Hence, a diketothioester is formed, which results in chain interconnection and the release of the catalytic cysteine. Subsequently, lactonization can take place. It is assumed that an enolate exists as an intermediate in the formation of the C–O bond [88]. Even though for both enzymes no experimental evidences for the chronological order of the two condensation reactions exist, it can be expected that the C–C bond is formed prior to lactonization [88]. For the following lactonization process a spontaneous reaction can be anticipated, which takes place once the two chains are interconnected, since thereby the atoms needed for lactonization are positioned in close proximity to each other. The sterical requirements within the catalytic cavity of CorB and MxnB do not favor the ring closure, thus the second step might take place in solution [90].

It has to be mentioned that the results between CorB and MxnB differ slightly. The in vitro results obtained for MxnB imply that the western chain gets covalently attached, prior to condensation with the second chain. The transfer of the western chain from the corresponding ACP to MxnB occurred much faster than the transfer of the eastern chain [88]. However, concerning CorB it was possible to observe a substantial positive electron density at the catalytic cysteine as a result of substrate incubation prior to crystallization. This was only possible with a very short substrate mimic which renders more similarity to the eastern chain. Using the longer western chain mimic no suitable crystals for structure determination could be produced (neither for CorB, nor for MxnB). Thus, in the CorB model the eastern chain was covalently attached. These inconsistent results indicate that the use of different chains could alter the binding preference.

**Figure 24:**
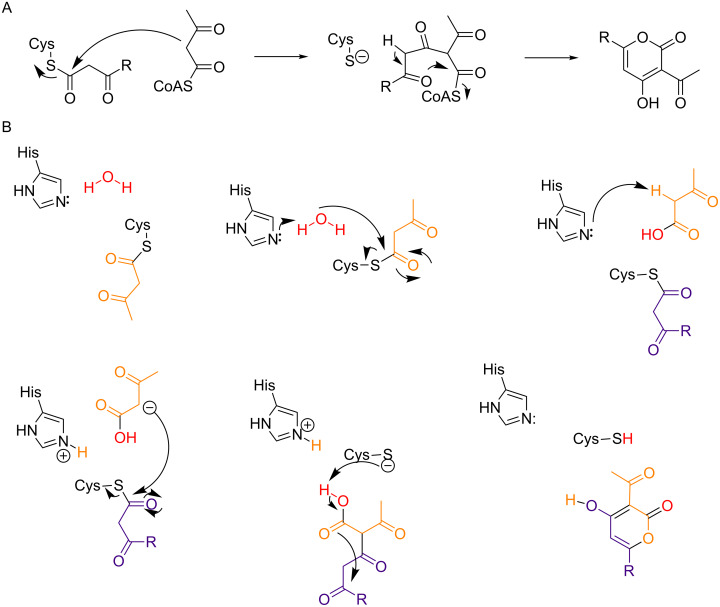
Proposed mechanism for the CsyB enzymatic reaction. A) Coupling reaction of the β-keto fatty acyl intermediate with acetoacetyl-CoA followed by pyrone ring formation (modified from [85]). B) Detailed mechanism; the two chains are color coded (orange and violet), as well as the water molecule (red) whose oxygen atom is incorporated into the α-pyrone (modified from [91]).

Also CsyB from *Aspergillus oryzae* catalyzes the condensation of two β-ketoacyl-CoAs [85]. However, this mechanism to form 3-acetyl-4-hydroxy-6-alkyl-α-pyrones (**79–81**) significantly differs from the one catalyzed by the myxobacterial ketosynthases described before [89]. CsyB is indeed an up to now unexemplified case of a type III PKS with dual function. First, CsyB catalyzes chain elongation – as many other PKS III enzymes. Secondly, it catalyzes the condensation of two β-ketoacyl units – a mechanism comparable to the enzymes described in the previous paragraph. It possesses two β-ketoacyl-CoA coupling activities to synthesize acylalkylpyrone. The initially proposed mechanism for the formation of 3-acetyl-4-hydroxy-6-alkyl-α-pyrone by CysB was the coupling of a β-keto fatty acid acyl intermediate with acetoacetyl-CoA, followed by pyrone ring formation (Figure 24 A) [85]. Then, as the crystal structure was solved the authors proposed the detailed mechanism as follows [91]: First, acetoacetyl-CoA is loaded onto the catalytic cysteine residue. Subsequently, the thioester bond is cleaved by the nucleophilic water molecule, which itself is activated through hydrogen bonding to the catalytic cysteine and a histidine residue. Thereby, the β-keto acid intermediate is generated. This intermediate is proposed to be placed within the novel pocket, a cavity accessible from the conventional elongation/cyclization pocket. After the replacement of the first β-keto acid, the second β-ketoacyl unit is produced. The catalytic cavity of CysB is loaded with a fatty acyl-CoA which is elongated with one molecule of malonyl-CoA, yielding the second β-ketoacyl chain. Condensation of the two chains generates the final product, whereby first the two chains are interconnected due to a nucleophilic attack, and subsequently an intramolecular lactonization takes place. In that way the ring closure results in the elimination of a water molecule, yielding the csypyrones harboring four *O*-atoms. The first step of the proposed mechanism was delignated from a set of in vitro assays, which indicated that the ^18^O atom of the H_2_^18^O molecule – which should be activated by hydrogen bonds networks with a histidine and the catalytic cysteine residue – is enzymatically incorporated into the final product (Figure 24 B). However, this mechanism is hard to prove, because ^18^O incorporation into the molecule can occur due to spontaneous exchange. Anyway, CysB clearly differs from CorB and MxnB. The latter condense two β-ketoacyl chains in a Claisen-like reaction to form the α-pyrone, while CysB should first generate a β-keto acid intermediate by hydrolysis of the thioester bond. Then the starter of the second chain is loaded onto the free catalytic cysteine, gets elongated by a malonyl-CoA before the nucleophilic attack of the first chain. In that way the thioester bond is cleaved and subsequently lactonization takes place, yielding in the final product (Figure 24 B).

In *Photorhabdus luminescens* it was shown that α-pyrones act as bacterial signaling molecules at low nanomolar concentrations [14]. A similar mechanism for the biosynthesis of these photopyrones as for the above mentioned α-pyrone antibiotics myxo- and corallopyronin was expected. To identify the gene corresponding to the biosynthesis of these so-called photopyrones, all ketosynthases which are not part of the usual fatty acid biosynthesis had been identified in the genome of *P. luminescens.* Thereby the ketosynthases neighbored by genes related to fatty acid synthesis had not been considered. Insertion mutants were generated and the influence on photopyrone production was analyzed. Thus, the gene *ppyS* (for photopyrone synthase) was identified, since all other disruption mutants did not yield in a photopyrone negative strain. Heterologous expression of *ppyS* in *E. coli*, together with the *bkdABC* operon (encoding the branched chain α-ketoacid dehydrogenase (Bkd) complex) and *ngrA* (encoding a phosphopantetheinyl-transferase which is essential to generate the *holo*-acyl carrier protein BkdB) for the biosynthesis of branched-chain iso-fatty acid, resulted in the production of photopyrone derivatives. This was a functional proof that PpyS catalyzes the formation of α-pyrones, as indicated before by feeding experiments with stable isotope-labeled precursors. PpyS should connect 5-methyl-3-oxohexanoyl thioester and different thioesters of straight-chain and iso-branched chain fatty acids [14]. The mechanism proposal also includes the catalytic cysteine. The first chain, i.e., thioester-activated 9-methyldecanoic acid, gets covalently tethered to that important residue within the active site. This reflects the same mechanism as for the other KS-like enzymes described. Also for PpyS the proposal postulates that the α-carbon of the enzyme-bound chain acts as a nucleophile. Thus, this activated carbon executes a nucleophilic attack on the carbonyl carbon of chain two, i.e., 5-methyl-3-oxohexanoyl thioester, which is itself synthesized by the Bkd complex. In that way a C–C bond is formed, and both chains are still attached to the catalytic cysteine residue. This bound intermediate undergoes a further deprotonation, which enables the formation of the α-pyrone ring. Through the ring closure the α-pyrone is released from PpyS. This second deprotonation can occur spontaneously, or enzyme catalyzed. In contrast to the cases of myxopyronins **36** and **37** and corallopyronins **34** and **35**, no PKSI system provides the ACP-bound chains. Therefore, the substrates for the chain interconnection might be either ACP or CoA bound. This would be depending on their origin in the cell, either fatty acid biosynthesis or degradation. The flexibility of the system in regard to the first chain to be bound to PpyS was already shown by the photopyrones A–H, which differ in the chain length and in the either branched or unbranched starting unit.

**Figure 25:**
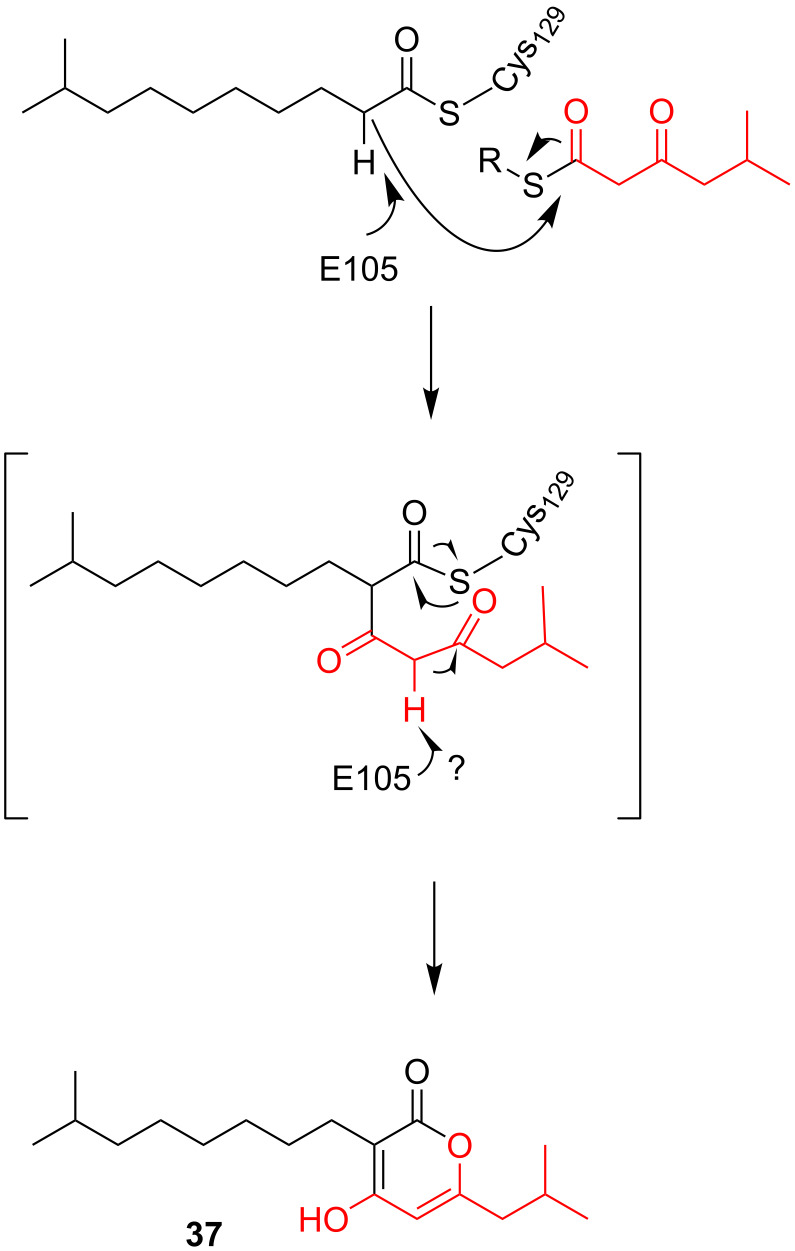
Proposed biosynthesis of photopyrone D (**37**) by the enzyme PpyS from *P. luminescens* (modified from [63]). The catalytic cysteine and the glutamate residue postulated to be involved in the biosynthesis are indicated. The two chains are colored in red and black, respectively.

No crystal structure for PpyS exists. Therefore, the structure was modeled using OleA from *Xanthomonas campestris*, which is showing the highest sequence identity (27%) of all available PDB-deposited crystal structures as template. Using the generated homodimeric model of PpyS, docking studies of the substrates onto the catalytic cysteine were performed. The resulting model suggested that a glutamate residue, which reaches into the catalytic cavity of the respectively other homodimer, acts as a base by forming a hydrogen bond with the α-carbon of the covalently bound substrate (Figure 25). Indeed, the exchange of this glutamate against an alanine residue resulted in an inactive version of the protein. Further an arginine residue, which could be involved in dimerization, was mutated to an aspartate. Also this mutant lost its catalytic activity, indicating that dimerization is essential [63].

The pseudopyronine synthase PyrS represents a homologue of PpyS. Using PpyS from *Pseudomonas* sp. GM30, it was analyzed if this KS is also involved in the formation of α-pyrones. The two pseudopyronines A (**55**) and B (**56**) have been up to now isolated from different *Pseudomonas* strains. Recently, in an independent publication **55** and **56** have been rediscovered from the banana rhizobacterium *Pseudomonas putida* BW11M1 [86]. Feeding studies with isotopically labelled precursors supported the biosynthesis from two chains. Subsequent analysis of the draft genome of the strain revealed a *ppyS* homologue. However, instead of the syntenic genomic region where pseudomonads usually harbor the *ppyS* homologue, it appeared that the gene has inserted between genes belonging to carbohydrate metabolism in *P. putida* BW11M1. An in-frame deletion mutant of the *ppyS* homologue was constructed and yielded in a strain which lost the opportunity for pseudopyronine biosynthesis [86]. Despite the similar mechanism for α-pyrone formation by PpyS homologues in the different *Pseudomonas* strains, a phylogenetic analysis revealed that different clades of PpyS exist. These different clades reflect also different locations in the genome sequences of the different *Pseudomonas* species: On a taxonomic level closely related strains harbor the *ppyS* homologue in the same region of their genome. Therefore, it can be assumed that the genetic information coding for the enzyme needed to synthesize pseudopyronines was acquired several times. Hence, Pseudomonas species from different habitats, e.g., rhizosphere, soil, water, acquired the gene set independently [86].

In summary different types of chain-interconnecting KSs which catalyze α-pyrone ring formation were identified in the last years. One mechanism is to fuse two ketoacyl moieties, as exemplified by CorB and MxnB. Another mechanism is the fusion of one ketoacyl moiety with one acyl moiety, as shown for PpyS-like KSs. All evolved from FabH-type KSs, but form different clades in phylogenetic analyses. PpyS-like enzymes show the conserved glutamate residue – indicating a mechanism distinct from the ketoacyl–ketoacyl-connecting KSs – and were identified in different bacterial genera, i.e., *Burkholderia, Legionella, Nocardia, Microcystis* and *Streptomyces*, therewith also in clinically relevant pathogens [63]. Future work will reveal which natural products are biosynthesized by such KSs, and which relevance these products have.

## Conclusion

The α-pyrones show an extraordinary wide variation in biological activities, independently if structurally simple or complex, naturally or non-naturally synthesized. Therefore, α-pyrones represent a rich source for isolation studies and lead discovery. Now, new insights into the biosynthesis of these molecules through chain interconnecting ketosynthases were obtained. This opens up the possibility to use these enzymes as tools; both, in bio- as well as in semi-synthetic approaches. The potential of these enzymes in combinatorial biosynthesis has to be further evaluated in the future.
